# Community-Acquired Respiratory Distress Syndrome Toxin: Unique Exotoxin for *M. pneumoniae*

**DOI:** 10.3389/fmicb.2021.766591

**Published:** 2021-11-19

**Authors:** Xiaoling Su, Xiaoxing You, Haodang Luo, Keying Liang, Li Chen, Wei Tian, Zufeng Ye, Jun He

**Affiliations:** ^1^The Affiliated Nanhua Hospital, Department of Clinical Laboratory, Hengyang Medical School, University of South China, Hengyang, China; ^2^Institute of Pathogenic Biology, Hengyang Medical School, Hunan Provincial Key Laboratory for Special Pathogens Prevention and Control, Hunan Province Cooperative Innovation Center for Molecular Target New Drug Study, University of South China, Hengyang, China

**Keywords:** community acquired respiratory distress syndrome toxin, *Mycoplasma pneumoniae*, ADP-ribosyltransferase, vacuolization, asthma

## Abstract

*Mycoplasma pneumoniae* infection often causes respiratory diseases in humans, particularly in children and adults with atypical pneumonia and community-acquired pneumonia (CAP), and is often exacerbated by co-infection with other lung diseases, such as asthma, bronchitis, and chronic obstructive pulmonary disorder. Community-acquired respiratory distress syndrome toxin (CARDS TX) is the only exotoxin produced by *M. pneumoniae* and has been extensively studied for its ADP-ribosyltransferase (ADPRT) activity and cellular vacuolization properties. Additionally, CARDS TX induces inflammatory responses, resulting in cell swelling, nuclear lysis, mucus proliferation, and cell vacuolization. CARDS TX enters host cells by binding to the host receptor and is then reverse transported to the endoplasmic reticulum to exert its pathogenic effects. In this review, we focus on the structural characteristics, functional activity, distribution and receptors, mechanism of cell entry, and inflammatory response of CARDS TX was examined. Overall, the findings of this review provide a theoretical basis for further investigation of the mechanism of *M. pneumoniae* infection and the development of clinical diagnosis and vaccines.

## Introduction

Mycoplasma are cell wall-less and self-replicating prokaryotic microorganisms capable of causing diseases in animals and plants (Baseman et al., 1995; Rottem, 2003). *Mycoplasma pneumoniae* is one of the most prevalent atypical bacterial respiratory pathogens that causes human infections associated with the community-acquired pneumonia (CAP) and accounts for up to 40% of CAP in children over 5 years of age (Atkinson et al., 2008; Lee, 2008; Youn and Lee, 2012; Atkinson and Waites, 2014). Additionally, *M. pneumoniae* is considered to be the causative agent of acute and chronic airway-related inflammations, such as tracheobronchitis, asthma, and chronic obstructive pulmonary disease, and extrapulmonary diseases (Berg et al., 2009; Watanabe et al., 2014; He et al., 2016; Kumar et al., 2019).

*M. pneumoniae* was initially isolated in 1962, and contains 816 kb chromosome, which encodes approximately 694 proteins in length with a G+C content of approximately 40% (Waites and Talkington, 2004; Lluch-Senar et al., 2015a; Xiao et al., 2015). The cell membrane of *M. pneumoniae* is one of the most important structures for its survival, which is a three-layer membrane composed of inner and outer layers of proteins and polysaccharides and a middle layer of lipids. The functions of the cell membrane includes adhesion, pathogenesis, absorption, metabolism, respiration, and maintenance of cell integrity.

Historically, it was found that the human lung protein surfactant protein A (hSP-A) can bind to a certain protein of *M. pneumoniae*. This protein sequence was identified with the putative protein MPN372 through purification and expression techniques, and subsequently named the community-acquired respiratory distress syndrome toxin (CARDS TX) (Kannan et al., 2005; Kannan and Baseman, 2006). CARDS TX has been shown to possess two properties: ADP-ribosyltransferase (ADPRT) activity and cellular vacuolization (Kannan and Baseman, 2006). CARDS TX can independently cause cilia stagnation, vacuolization, nuclear fragmentation, and the release of inflammatory factors in infected mammalian cells, similar to the cytopathic pathology induced by *M. pneumoniae*. CARDS TX shares high similarity with the pertussis toxin S1 subunit (Hardy et al., 2009). CARDS TX, cytoadherence, and the production of hydrogen peroxide and hydrogen sulfide are the major pathogenicity determinants underlying the ability of *M. pneumoniae* to cause human disease (Großhennig et al., 2016). Importantly, CARDS TX not only possesses a high immunogenic response *in vivo* and *in vitro*, but also mediates cells infection in a dose-, temperature-, and time-dependent manner (Kannan et al., 2005; Kannan and Baseman, 2006; Hardy et al., 2009; Muir et al., 2011). This review focused on the structures and features of CARDS TX, a unique exotoxin produced by *M. pneumoniae*, which is responsible for acute or chronic infections.

## Structures and Features of Community-Acquired Respiratory Distress Syndrome Toxin

### Protein Sequences of Community-Acquired Respiratory Distress Syndrome Toxin

Primarily, CARDS TX is a 68-kDa protein consisting of 591-amino-acid, and exhibits the ADPRT activity and the cytoplasmic vacuolization (Kannan et al., 2005; Kannan and Baseman, 2006; Pakhomova et al., 2010). The N-terminal of CARDS TX possessed both mono-ADP-ribosyltransferase and NAD^+^-binding activity, whereas the C-terminal binds to receptors, internalization, and vacuolation activity compared with full-length and truncation variants of CARDS TX (Kannan et al., 2014; Becker et al., 2015). The N-terminal domain possesses three conserved motifs, including a conserved arginine, a serine-threonine-serine (STS) motif, and a catalytic glutamate, which play essential roles in ADPRT activity (Becker et al., 2015). Moreover, the N-terminal of CARDS TX also binds to CARDS toxin-specific immunoglobulin E (IgE) (Medina et al., 2017).

Notably, it has been demonstrated that annexin A2 (AnxA2) was mainly bound to the C-terminal of CARDS TX in the interaction of CARDS TX-host cells (Somarajan et al., 2014). The C-terminal region of the CARDS TX has better sensitivity and specificity in human sera than the full-length and N-terminal region as indicated by enzyme-linked immunosorbent assays (ELISA) (Xue et al., 2021). Compared with the previous studies of recombinant P1, P30, and Mpn456 proteins, the C-terminus of the CARDS TX showed better sensitivity and specificity, enhancing the binding of the receptors to the host cell membrane (Xue et al., 2021). In contrast, the expression of CARDS TX devoid of the N-terminal domain in *M. pneumoniae* caused a decrease in vacuolization of mammalian cell lines during infection, indicating an important role of N-terminus in maintaining the conformational integrity of the C-terminus (Kannan et al., 2014). Further research is needed to determine whether the recombinant CARDS TX (rCARDS TX) antigen is the best indicator for *M. pneumoniae* in clinical testing.

### Structure of Community-Acquired Respiratory Distress Syndrome Toxin

CARDS TX is comprised of a three-domain structure: domain 1 (D1) at the N-terminus, domain 2 (D2), and domain 3 (D3) at the C-terminus, and it includes 17 α-helices and 43 β-strands (Kannan and Baseman, 2006; Pakhomova et al., 2010; Becker et al., 2015). The D1 domain, designated as the mono-ADP ribosyltransferase (mART) domain, is composed of residues 1–205, which possesses mART activity. Residues 206–256 are the NAD^+^-binding site and residues 257–272 are the linkers connecting D1 to D2+D3. The tandem D2 domain (residues 273–439) and D3 domain (residues 440–591) form β-trefoil domains, which are responsible for the internalization and the vacuolating activity (Becker et al., 2015). The interface where between D1 and D2+D3 are connected is very broad and mainly polar, which is easily broken (Becker et al., 2015). Perhaps the cleavage of the CARDS TX is followed by a break from the interface before loss of activity; however, this is subject to further study.

Generally, sequence alignment and structural analysis revealed that CARDS TX contains several conserved structural motifs that mediate its function. For instance, the mART domain of CARDS TX primarily exhibits the ADPRT activity. For example, the unique R–STS–E motif (R10, S49, T50, S51, and E132) contributes to the NAD^+^ binding and the transferase activity (Kannan and Baseman, 2006). The conserved arginine (R10) and the Ser-Thr-Ser (S49-T50-S51) motifs at the N-terminus are believed to interact with the NAD^+^ cofactor. The invariant catalytic Glu (E132) is responsible for the transferase activity and the ADP-ribosylating turn-turn (ARTT) motif (S126-F134) is implicated in substrate specificity and protein-protein recognition. The helix-strand-helix motif (α4-β6-α5) is involved in an important interaction between the D1 and D3 domains (Becker et al., 2015). Additionally, CARDS TX induces ADP-ribosylation of the NLRP3 inflammasome (Bose et al., 2014), indicating that the mART domain might play a crucial role in CARDS TX-inflammasome interaction. Losing the last 20 residues (residues 571–591) of CARDS TX inhibited the internalization activity. Residues 571–591 are indispensable for the proper folding and formation of aromatic patches from the D3 domain (Becker et al., 2015). Aromatic patches are related to cell surface binding and internalization (Becker et al., 2015). Therefore, it is reasonable to assume that the D3 domain plays an important role in internalization activities (Becker et al., 2015; Ramasamy et al., 2018). Notably, the vacuolating activity is regulated by D2+D3 and is not affected by the D1 domain (Kannan et al., 2014; Becker et al., 2015).

**FIGURE 1:**
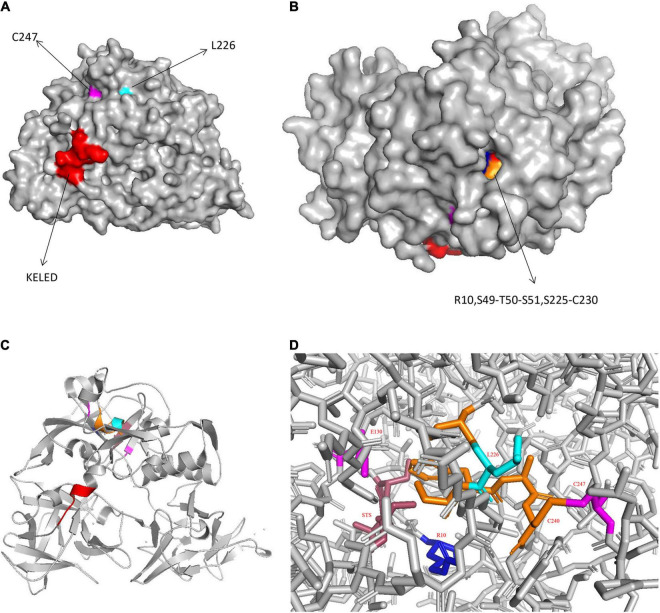
Structure of CARDS TX. **(A)** Surface representation of CARDS TX with its signature motifs. **(B)** Invaginated expression sites of CARDS TX. **(C)** Cartoon representation of CARDS TX. **(D)** The stick model of CARDS TX. (Red: KELED motif; blue: R10; magenta: E130 and C247; orange: S225-C230; cyan: L226).

Interestingly, CARDS TX contains a ^268^KELED^272^ motif, which is similar to the KDEL endoplasmic reticulum retention motif that mediates the protein trafficking from the Golgi complex to the endoplasmic reticulum (ER). Furthermore, the KDEL motif contains a Lys-Asp-Glu-Leu signal, which plays an essential role in its retrograde transport, and is often located at the C-terminus of other bacterial toxins, such as cholera toxin B subunit from *Vibrio cholerae* (Ramasamy et al., 2018; Royal et al., 2019). The KELED motif of CARDS TX is located at the solvent-accessible linker region between the D1 and D2 domains. The solvent-accessible surface area of proteins has always been considered as a decisive factor in protein folding, stability, and protein and ligand binding free energy studies (Ali et al., 2014), suggesting that the KELED motif might influence the function of protein folding and stability (Figure 1).

### Protection of Disulfide Bridge in the Community-Acquired Respiratory Distress Syndrome Toxin

Generally, the disulfide bridge plays a role in maintaining the stability of toxin proteins from bacterial, including diphtheria toxin, botulinum, tetanus neurotoxins, and apoptosis-inducing protein from *Photobacterium damselae* subsp (Balasubramanian et al., 2019; Landeta et al., 2019; Pereira et al., 2020). Previous studies have shown that thiol-reducing reagents such as dithiothreitol (DTT), can activate CARDS TX by reducing and cleaving of disulfide bonds, thereby increasing ADP-ribosylation activity (Kannan and Baseman, 2006). This result suggests that the CARDS TX undergoes conformational changes, exposing the active site to improve substrate binding (Kannan and Baseman, 2006).

CARDS TX contains six cysteine residues (C230, C247, C324, C406, C425, and C548) that are likely to form disulfide bridges. However, only C230 and C247 form an intramolecular disulfide bond at the N-terminal domain in CARDS TX (Balasubramanian et al., 2019). The disulfide bond between C230 and C247 could stabilize residues 225–230 (α9) from the NAD^+^-binding site. Moreover, there is a significant decrease in the expression of interleukin-1β (IL-1β) in C230S (C→S mutant) toxin compared with the wild-type (WT) CARDS TX (Balasubramanian et al., 2019). The broken disulfide bond could cause a release in ADP activity, indicating the essential role of the disulfide bridge in NLRP3 ADP-ribosylation and inflammasome activation to release cytokine IL-1β. The formation of disulfide bonds protects the CARDS TX mART domain from proteolysis by proteases such as trypsin, thermolysin, and proteinase K. Moreover, the disulfide bond is critical for proper execution of ADPRT activity of CARDS TX. Additionally, results showed that C230S toxin did not elicit vacuole formation in U937 cells but recovered its vacuolating activity after protease was added. Although the disulfide bond of CARDS TX is dispensable for cell binding, internalization, and intracellular trafficking, vacuolating activity is inhibited in mutated CARDS TX lacking the disulfide bond inhibits (Balasubramanian et al., 2019). Overall, the disulfide bond plays a crucial role in ADPRT activity and the subsequent cytopathological phenomena of CARDS TX.

Furthermore, it is appreciated that the disulfide bond is also crucial for maintaining the stability of the CARDS TX and it needs to be intact for efficient translocation of CARDS TX into the cytosol (Balasubramanian et al., 2019; Landeta et al., 2019; Pereira et al., 2020). Overall, the disulfide bond may be used as a potential target for the development of vaccines for *M. pneumoniae*.

### Community-Acquired Respiratory Distress Syndrome Toxin Homologues

**FIGURE 2:**
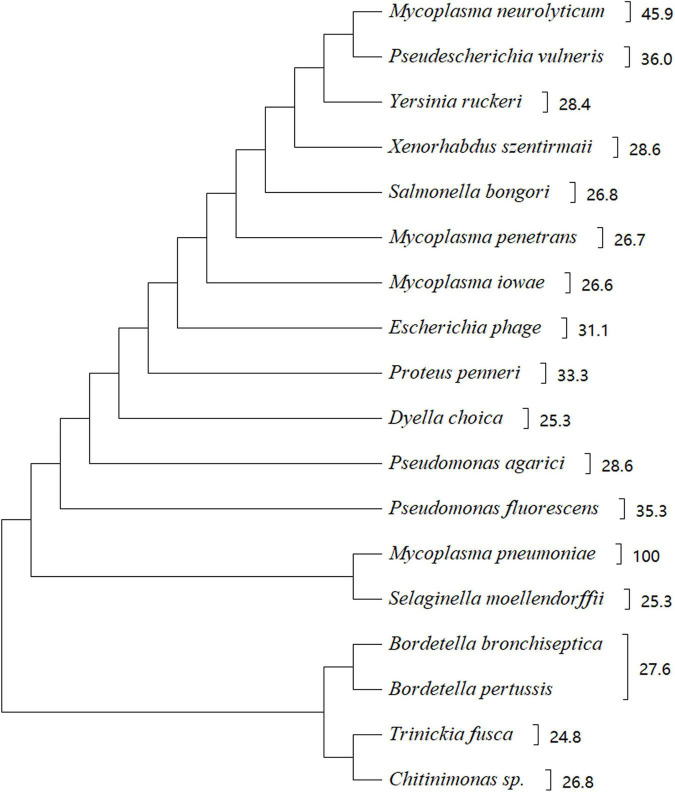
Phylogenetic tree showing the homology between CARDS TX and other bacterial proteins. There were 18 strains with a score of 110 or more selected from https://www.uniprot.org/blast. The phylogenetic tree shows 18 species of microorganisms that are highly homologous to CARDS TX, such as *Mycoplasma neurolyticum, Mycoplasma iowae, Mycoplasma penetrans*, and others. CARDS TX of *M. pneumoniae* is highly homologous with ADP-ribosylating toxin CARDS TX of *M. neurolyticum*. The evolutionary history of CARDS TX was examined using the Maximum Parsimony method. Evolutionary analyses were conducted using MEGA X software based on the BLAST web page of protein sequence.

*In silico* analysis of amino acid sequences indicated that CARDS TX shares high sequence similarity with other bacterial toxins, including the pertussis toxin S1 subunit from *Bordetella pertussis*, cholera toxin (CT) from *V. cholerae*, diphtheria toxin (DT) from *Corynebacterium diphtheriae*, and similar CARDS TX homologues protein from *Mycoplasma neurolyicum, Mycoplasma iowae*, and *Mycoplasma penetrans* (Figure 2). Sequence alignment indicated that the pertussis toxin S1 subunit exhibited 27% identity and 41% similarity with the N-terminus (residues 1–239) of CARDS TX. The N-terminus of the hypothetical protein MYPE9110 from *M. penetrans* (residues 22–214) shares higher homology (27% identity and 45% similarity) with CARDS TX from *M. pneumoniae*. In contrast, the C-terminus of MYPE9110 (residues 485–624) shares, 23% identity and 40% similarity than CARDS TX (Johnson et al., 2009). Notably, MYPE9110 can still exhibit the ADPRT activity in the absence of the Ser-Thr-Ser (STS) motif and induce cytoplasmic vacuolization in the presence of ammonium chloride in HeLa cells (Johnson et al., 2009). Weak bases, such as ammonium chloride, can stimulate the MYPE9110 binding to HeLa cells, inducing the cytoplasmic vacuolization, similar to the molecular mechanism of VacA cytotoxin from *Helicobacter pylori* (Cover and Blanke, 2005).

Similarly, CARDS1 and CARDS2 from *M. iowae* serovar K share 25 and 28% sequence identity with *M. pneumoniae* CARDS TX, respectively. Although the potential activity of *M. iowae* has not been examined, genomic analysis and comparison showed that it may possess ADPRT-like activity (Pritchard and Balish, 2015). The expression of CARDS1 in *M. iowae* is reduced under low O_2_ conditions, and CARDS1 is associated with the reduced pathogenicity in the gut. Regarding CARDS TX from *M. pneumoniae*, the role of O_2_-mediated inactivation of CARDS TX in pathogenicity remains unclear. Interestingly, *M. genitalium* has the highest affinity for *M. pneumoniae*, but *M. genitalium* does not express the protein with high homology similar to CARDS TX from *M. pneumoniae*.

### Variation of Community-Acquired Respiratory Distress Syndrome Toxin in Different *M. pneumoniae* Strains

It has found that nucleotide polymorphism in CARDS TX from four clinical strains designated L2, J1, RJL1, and S1, which changed in amino acids at certain positions in contrast to reference strain M129 (Kannan and Baseman, 2006). Strain S1 was the most variable, with a total of four variant loci. The amino acid position 371 (Ile to Ser) was changed in these clinical isolate strains (Kannan and Baseman, 2006). Major variation occurred in the P1 and ORF6 genes associated with the adhesin complex in *M. pneumoniae.* In contrast to P1 and ORF6, the CARDS TX gene has minimal variation among strains and is more conserved (Xiao et al., 2015).

Furthermore, studies have shown considerable increase in the concentration of CARDS TX and inflammatory factors, such as interferon-gamma (IFN-γ), IL-12, IL-1α, in bronchoalveolar lavage (BAL) of mice infected with *M. pneumoniae* strain S1 compared with those of mice infected with two other *M. pneumoniae* strains M129-B7 and M129-B9 (Techasaensiri et al., 2010), implying that strain S1 has a greater effect on bronchoalveolar lavage or lung histopathology. The clinical isolates of *M. pneumoniae* were divided into two main groups, types 1 and types 2, according to the differences in the P1 adhesin gene sequences (Lluch-Senar et al., 2015b). The M129 was found to be a type 2 strain of *M. pneumoniae*, whereas S1 is a type 1 strain (Kannan et al., 2010; Lluch-Senar et al., 2015b; Xiao et al., 2015). A previous study has found that the expression levels of CARDS TX in type 2 strains were higher than those in type 1 strains of *M. pneumoniae* (Lluch-Senar et al., 2015b). Moreover, it has been noted that type 2 strains are more toxigenic than type 1 (Lluch-Senar et al., 2015b; Feng et al., 2020).

Additionally, the formation of biofilms has been reported in type 2 strains, which are common factors responsible for persistent infections, antibiotics resistance, and immune evasion (Costerton et al., 1999; Simmons et al., 2013). Moreover, type 2 strains form more robust biofilms than type 1. However, there was a disproportional decrease in CARDS TX levels as biofilms mature (Feng et al., 2020). These findings may be why type 2 strains are more likely to cause epidemic infections than type 1 strains. However, whether mutation of CARDS TX in *M. pneumoniae* will affect the pathogenicity is yet to be examined. Moreover, future studies should examine the relationship between CARDS TX concentration and biofilm should be examined to determine whether it will affect biofilm maturation.

## Molecular Mechanism of Community-Acquired Respiratory Distress Syndrome Toxin in Pathogenicity

### Distribution and Expression of Community-Acquired Respiratory Distress Syndrome Toxin

*M. pneumoniae* adheres to the cytomembrane, causing inflammation effects and rupture of cells and tissues (Rottem, 2003; He et al., 2016; Parrott et al., 2016). According to immunogold labeling and electron microscopic analysis, CARDS TX is located on the entire surface the membranes of *M. pneumoniae*, including the tip organelle, similar to the P1, which is a major surface-associated adhesin protein (Kannan et al., 2010). Possibly, CARDS TX may mediate adherence in association with the tip organelle, facilitating contact between *M. pneumoniae* and host target cells (Kannan et al., 2010). Additionally, CARDS TX is more distributed in the cytoplasm of host cells (Kannan et al., 2010). Moreover, CARDS TX can bind to certain cell surface receptors and then spread within the host cells, eliciting the release of inflammatory factors that produce the cytopathic effect (Kannan et al., 2012; Atkinson and Waites, 2014). CARDS TX can be detected both on the respiratory epithelium cells and in the peribronchiolar alveolar spaces after infection (Kannan et al., 2012). Furthermore, it was implied that CARDS TX presents on the surface of the cilia of the epithelium, which normally expression co-localizes with mycoplasma cells that colonize respiratory epithelial cell surfaces after *M. pneumoniae* infection in mouse lungs (Berg et al., 2009). Notably, the intracellular distribution of CARDS TX acts in a temperature-time-dependent manner (Ramasamy et al., 2018).

Studies on the mRNA level of *cards* and the protein level of CARDS TX in *M. pneumoniae* S1 strain at different growth phases as well as in different cells or in SP-4 broth-grown cultures showed that there was a slight decrease in *cards* mRNA level at 12–24 h, followed by a sharp decrease at 24–48 h, and a gradual decrease thereafter. In contrast, CARDS TX protein levels in *M. pneumoniae* broth cultures peaked between 24 and 48 h followed by a rapid decrease over time, with the lowest level at 60 h (Hardy et al., 2009; Kannan et al., 2010; Feng et al., 2020). The expression profiles of *cards* mRNA and CARDS TX protein are non-linear, indicating that CARDS TX may be transcribed by another gene. Notably, the expression profile of CARDS TX protein was different from those of other proteins of *M. pneumoniae* such as P1 and P30 (Kannan et al., 2010). Typically, *M. pneumoniae* CARDS TX is only expressed at higher levels in host cells during the early-mid period of infection, whereas its expression is poor in SP-4 broth medium (Kannan et al., 2010), suggesting that CARDS TX protein synthesis is higher in *M. pneumoniae* cells *in vivo* than *in vitro*. Additionally, there was a gradual increase in the expression of CARDS TX per mycoplasma genome in mouse lung tissues at 24–48 h post-infection. The host cells establish an environment conducive for the survival of *M. pneumoniae* or CARDS TX. Alternatively, it was reported that the concentrations of CARDS TX, as well as the production of H_2_S and H_2_O_2_ were highest during early biofilm formation (48–72 h) and decreased over time (72–120 h) (Feng et al., 2020). Furthermore, CARDS TX level in bronchoalveolar lavage fluid (BALF) of infected mice was highest observed at 1 day post-infection and gradually decreased over time. However, although at a low level, CARDS TX was still detectable at 35 days post-infection (Kannan et al., 2011). Knowledge of the dynamics of toxin expression studied in different cells and tissues is necessary to better understand the inflammatory and pathological changes caused by CARDS TX or *M. pneumoniae*.

Moreover, serum IgM levels against CARDS TX and P1 peaked at 7 days post-infection in most experimental mice and was at the lowest level at 35 days post-infection. However, serum IgG levels against CARDS TX and P1 increased significantly between 7 and 35 days post-infection, according to seroconversion to *M. pneumoniae* CARDS TX and adhesin P1 in infected mice (Kannan et al., 2011). Furthermore, ELISA showed that serum IgM and IgG antibodies were more reactive against CARDS TX than P1 (Peters et al., 2011). Overall, serological analysis showed that animals exposed to CARDS TX had better IgM and IgG responses than animals infected with *M. pneumoniae*, indicating that CARDS TX has a high immunogenic (Kannan et al., 2011; Peters et al., 2011; Maselli et al., 2018). These findings further confirm the fact that CARDS TX can act as the main virulence factor in *M. pneumoniae* (Kannan et al., 2010).

### Community-Acquired Respiratory Distress Syndrome Toxin Interactions With Host Cell Receptors

Previous studies have suggested that CARDS TX can bind to the mammalian cell surface receptors such as surfactant protein-A (SP-A), annexin A2 (AnxA2), and non-proteinaceous receptors [phosphatidylcholine (PC), and sphingomyelin (SM)], which play an important role in cytopathology (Kannan et al., 2005; Kannan and Baseman, 2006; Somarajan et al., 2014; Becker et al., 2015). SP-A is considered the first barrier in the innate defense mechanism of lungs, and is not only distributed in alveolar type II cells, but also in non-pulmonary sites (Becker et al., 2015; Dy et al., 2018; Kumar et al., 2019). Interestingly, although SP-A can directly inhibit or kill the bacteria, it mainly produces bacteriostatic activity against *M. pneumoniae* (Piboonpocanun et al., 2005). SP-A can combine with two ligands from *M. pneumoniae* involved in different roles. Moreover, SP-A can interact with disaturated phosphatidylglycerol membrane surface lipids of *M. pneumoniae* that protected mucin against producing and neutrophil recruitment and then reduced the growth and pathogenicity of *M. pneumoniae* (Piboonpocanun et al., 2005; Großhennig et al., 2016; He et al., 2016). Importantly, studies have provided strong evidence that CARDS TX specifically interacts with SP-A in a dose-dependent and calcium (Ca^2+^)-dependent manner, that has high affinity (Kannan et al., 2005; Kannan and Baseman, 2006; Dy et al., 2018). However, more evidence is needed to identify which protein dominates the binding of *M. pneumoniae* to SP-A, which could improve the understanding of *M. pneumoniae*-host cell interaction.

Knocking down of SP-A in host cells did not affect the binding and vacuolating activities of CARDS TX, suggesting that alternative receptors exist on the cytomembrane (Kannan and Baseman, 2006). Research findings have shown that CARDS TX also binds selectively to AnxA2 in a concentration-dependent and saturable manner (Somarajan et al., 2014). Surprisingly, the AnxA2_267_ (residues 1–267) variant showed the maximum binding to CARDS TX compared to the other truncated derivatives of AnxA2, indicating that AnxA2_267_ may cause an increase in CARDS TX-interactive exposure sites, or that the remaining amino acid residues could inhibit the binding activity of AnxA2 to CARDS TX (Somarajan et al., 2014). Moreover, the C-terminus of CARDS TX is mainly mediated by interaction with AnxA2, confirming the binding activity of the C-terminus.

Analysis of the transport mechanisms of CARDS TX and AnxA2 by confocal microscopy and immunofluorescence indicated that CARDS TX was only detected on the surface of cell membranes whereas AnxA2 was present on the cytomembrane as well as in the cytoplasm at 4°C; however, CARDS TX was transferred into the cytoplasm at 37°C. It was proposed that CARDS TX colocalizes with AnxA2 first on the cytomembrane and then in the cytoplasm (Somarajan et al., 2014). Importantly, AnxA2, a member of the annexin family, is a multifunctional protein involved in various functions such as exocytosis, endocytosis, trafficking, and post-translational modifications, and is expressed on the surface of various eukaryotic cells, including epithelial and endothelial cells (Grindheim et al., 2017; Dallacasagrande and Hajjar, 2020). When temperature is increased to 37°C, CARDS TX binds more tightly to AnxA2, thus improving transport and active receptor-mediated uptake. AnxA2 not only mediates connection with CARDS TX but also regulates the classical property of CARDS TX in ADP-ribosylation and vacuolation. Notably, AnxA2 enhances CARDS TX-induced cytoplasmic vacuolization in AnxA2-deficient cells (Somarajan et al., 2014). Suppression or knockdown of AnxA2 and SP-A can reduce CARDS TX binding and cytoplasmic vacuolization. There may exist a competitive relationship between SP-A and AnxA2, but SP-A is more abundant than AnxA2 on the cytomembrane of the lungs (Kannan and Baseman, 2006; Somarajan et al., 2014). Although SP-A may be more abundant, AnxA2 is more functional.

In addition to the two receptors mentioned above, CARDS TX also binds to membrane lipids, such as phosphatidylcholine (PC) and sphingomyelin (SM), which are predominantly distributed in the outer leaflet of the cellular membrane of host calls, and both PC and SM mainly combine with D2+D3 in CARDS TX (Becker et al., 2015). However, further studies are necessary to comprehensively examine transport mechanism and internalization of membrane receptor proteins of CARDS TX. Additionally, more evidence is needed to demonstrate the role played by receptor proteins in CARDS TX-induced cytopathic and inflammatory factor release.

### Endocytic Mechanisms of Community-Acquired Respiratory Distress Syndrome Toxin

Another question that remains to be answered is how CARDS TX enters host cells. CARDS TX enters host cells through a decisive step, which involves transferring the toxin into the cell to infect the host cells. Studies have shown that CARDS TX enters host target cells *via* receptor-clathrin-mediated endocytosis (Kannan et al., 2005; Krishnan et al., 2013; Somarajan et al., 2014).

**FIGURE 3:**
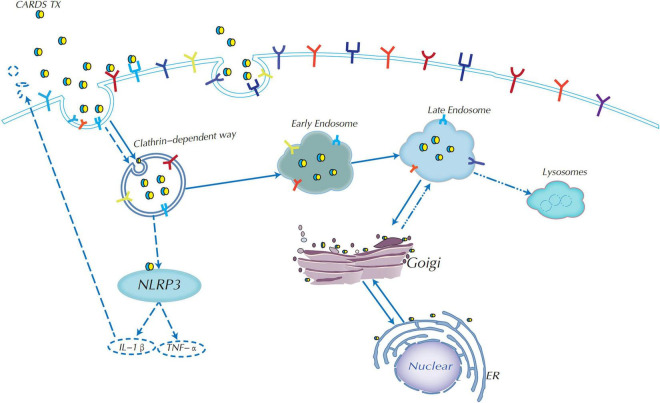
Endocytic mechanism of CARDS TX and signaling pathway diagram. CARDS TX could use receptor-clathrin-mediated endocytosis from early endosomes to late endosomes to enter host target cells. CARDS TX is trafficked by endoplasmic reticulum (ER) to the Golgi complex, most importantly, it could use Golgi complex retrograde transport to the ER in the help of the special KELED sequence, and subsequently transported outside the cell. CARDS TX could activate the NLRP3 inflammasome to release of IL-1β and associated pathologies.

Clathrin-mediated endocytosis (CME) is one of the most widespread and certainly the most well-studied internalization pathway for small viruses, bacteria, and toxins in recent years (McMahon and Boucrot, 2011; Krishnan et al., 2013; Khan and Steeg, 2021). Inhibition of the clathrin protein caused a decrease in CARDS TX internalization of HeLa cells, indicating that CARDS TX depends on the clathrin-mediated endocytosis pathway to enter host cells (Figure 3; Krishnan et al., 2013). Current studies have shown that vacuoles induced by rCARDS TX are acidic and derived from the endocytic pathway as Rab9 accumulates around vacuoles, whereas Rab9 is a small GTP-binding protein, and is a late endosomal marker (Johnson et al., 2011). Therefore, CARDS TX first needs to arrive at the late endosomes (Krishnan et al., 2013). It has been proposed that CARDS TX is transported from early endosomes to late endosomes, and then continuously trafficked by the endoplasmic reticulum (ER) to the Golgi complex *via* the ER-Golgi intermediate compartment (ERGIC) or by retrograde transport from the Golgi complex to the ER, and eventually transported outside the cell (Krishnan et al., 2013; Ramasamy et al., 2018). The mechanism of retrograde transport is activated by toxins containing a KDEL-like sequence, such as cholera toxin and Pseudomonas exotoxin A (Jackson et al., 1999; Sandvig et al., 2000). Recognition of the KDEL motif is performed by specific KDEL receptors. In mammalian cells, if the ER lacks the KDEL receptors, this cargo will be unable to return to the ER (Capitani and Sallese, 2009; Jia et al., 2021). Therefore, *M. pneumoniae* CARDS TX contains a novel KELED sequence similar to KDEL motifs, which play a crucial role in retrograde transport to the ER (Capitani and Sallese, 2009; Ramasamy et al., 2018). Additionally, single mutations in the E position of KELED (KALED and KELAD) did not significantly affect the functions of the CARDS TX, whereas mutation of the double E position (KALAD) significantly downregulated the transport activity of CARDS TX (Ramasamy et al., 2018). Moreover, the KALAD variant can cause conformation changes in CARDS TX, including IL-1β secretion and vacuolization in host cells. Overall, these findings indicated that the KALAD variant can inhibit CARDS TX retrograde trafficking to the ER, thereby preventing the activation of ADP-ribosylation of NLRP3, IL-1β release, and vacuolation cytopathy (Capitani and Sallese, 2009).

Moreover, deacidification of endosomes can significantly reduce the binding of the CARDS TX-Golgi complex, indicating that an acidic endosomal environment is essential for the retrograde transport of CARDS TX to the ER. Meanwhile, endosomal pH affects CARDS TX cleavage, but not binding and/or entry, whereas CARDS TX cleavage is required for tight attachment to the Golgi complex (Ramasamy et al., 2021). Overall, both KELED sequences and acidic endosomal environments play essential roles in retrograde transport and cytopathic effects (Ramasamy et al., 2018, 2021).

### Community-Acquired Respiratory Distress Syndrome Toxin and NLRP3 Activation

Inflammation is generally triggered by the innate immune responses during infection or tissue damage (Bose et al., 2014). NLRP3 (NLR-family, leucine-rich repeat protein 3) inflammasome is an important inflammasome complex that contains its central protein NLRP3, the adaptor protein ASC, the mitotic kinase NIMA-related kinase 7 (NEK7), and the effector protein caspase-1. NLRP3 can activate caspase-1, which can cleave pro-cytokines including pro-IL-1β and pro-IL-18 to transfer into its mature form (Shimizu, 2016; Groslambert and Py, 2018; Hooftman et al., 2020).

In contrast to wild-type cells, treatment of NLRP3 inflammasome-deficient mouse primary bone marrow-derived macrophages (BMDMs) with CARDS TX result in a considerable decrease in IL-1β expression (Bose et al., 2014). Studies have shown that CARDS TX can also exhibit ADPRT activity by activating the NLRP3 inflammasome through post-translational modification, thus catalyzing ADP-ribosylation of NLRP3 (Bose et al., 2014; Segovia et al., 2018). ADP-ribosylation is a unique post-translational modification process and an important covalent chemical modification process that is widely present in pathogenesis, intracellular signaling systems, DNA repair, and cell division (Han and Tainer, 2002). Retained ADPRT activity of mutant and/or truncated CARDS TX that was able to activate NLRP3 inflammasome making ADP-ribosylation of NLRP3, meanwhile, indicated that ADPRT amino acid motifs in the N-terminal of CARDS TX were important for activating NLRP3 inflammasome (Bose et al., 2014). Previous studies proposed that cytoadherence or lipid licensing of *M. pneumoniae* induces inflammatory responses through autophagy and toll-like receptor 4 (TLR4) to activate of NLRP3 inflammasome (Shimizu et al., 2014; Luo et al., 2021). These findings indicate that rCARDS TX can elicit IL-23 expression in human monocytes and RAW264.7 cell through TLR4 pathways (Wang et al., 2020). We speculate that TLR4 may play important roles in the process of CARDS TX-induced NLRP3 and autophagy. However, whether CARDS TX exerts its effects through other pathways is subject to further studies.

### Cytokine Expressions and Pathological Feature of Community-Acquired Respiratory Distress Syndrome Toxin

Administration of rCARDS TX to mouse and baboon BALF in a dose- and time-dependent manner caused a significant increase in the concentrations of pro-inflammatory factors such as IL-1α, IL-1β, IL-6, IL-12, IL-17, and TNF-α, and chemokines, including keratinocyte-derived chemokine (KC) and IL-8. However, there was an increase in IFN-γ expression only in the baboon but not in the mice (Hardy et al., 2009; Techasaensiri et al., 2010). In naive mice, rCARDS TX administration increased the expression of the Th-2 cytokines IL-4 and IL-13 as well as the Th-2 chemokines CCL17 and CCL22 (Medina et al., 2012). In baboons, rCARDS TX increased the ratio of IL-4/IFN-γ over time (Maselli et al., 2018). The expression of CARDS TX, TNF-α, and IL-6 in BALF was significantly increased in refractory *M. pneumoniae* pneumonia (RMPP) cases compared with non-RMPP (NRMPP). Subsequently, it was thought that high co-expression of TNF-α and CARDS TX is a good predictor for refractory *M. pneumoniae* pneumonia (Li et al., 2019). Whether the role of CARDS TX in *M. pneumoniae* inflammation-inducing factors plays a major role in the pathogenic effect is still worth investigating. The mechanism of exocytosis of the CARDS TX is not yet clear, and it needs to be investigated whether it is degraded intracellularly or whether it can infect other neighboring cells through certain pathways.

Morphological changes, including disruption of tissues integrity, disappearance of ciliary and microvilli motion, cytoplasmic vacuolization, nuclear swelling, and nuclear fragmentation, were observed by transmission electron microscopy within 48–72 h of culturing baboon tracheal organ rings with rCARDS TX (Kannan and Baseman, 2006). Under normal circumstances, cells were renewal and vacuolization disappeared at a certain point in time (Kannan and Baseman, 2006). Moreover, CARDS TX can also elicit changes within the pulmonary compartment or in airway function, which can cause peribronchial and perivascular lymphocytic infiltration of the bronchiolar epithelium. Intranasal instillation of rCARDS TX in mice caused airway obstruction (AO) and airway hyper-reactivity (AHR) in the mouse lungs tissue. Overall, these changes were similar to those reported for *M. pneumoniae* infection (Watanabe et al., 2014). Mice exposed to a single dose of rCARDS TX developed prolonged AO over 21 days and AHR at 2 days post-exposure. However, there was a decrease in AHR prior to resolution of AO, indicating that AHR requires sustained exposure to toxins, which is typical of the infectious process (Hardy et al., 2009). Subsequently, it was demonstrated that the rCARDS TX concentration and lung histopathological score (HPS) are positively correlated (Techasaensiri et al., 2010). Furthermore, there was an 85-fold increase in the Muc5AC (the major mucin protein) mRNA level of the lungs of rCARDS TX-treated mice compared with that of the control group (Medina et al., 2012). Additionally, previous studies have demonstrated that *M. pneumoniae* induces airway mucin expression, which is dependent on the activation of TLR2 receptor signaling, and excessive mucus metaplasia can aggravate asthma (Chu et al., 2005; Kraft et al., 2008). CARDS TX induced mucus metaplasia, and mast cell degranulation, and the accumulation of eosinophils and lymphocytes, indicating that rCARDS TX can induce allergic inflammation in naive animals and may be a pathogenic factor in *M. pneumoniae*–associated asthma (Medina et al., 2012, 2017). CARDS TX may depend on the activation of TLR2 receptor signaling to induce alterations in cytopathology.

Among patients infected with *M. pneumoniae*, asthma and other respiratory diseases are common in patients with respiratory barrier damage (Medina et al., 2014; Watanabe et al., 2014). Studies have confirmed that under prolonged ventilator treatment and hypoxemia, as well as in asthmatic patients, the positive rate of CARDS TX is higher than that of P1 protein. These findings indicate that CARDS TX may cause mycoplasma-induced asthma to destroy the cell barrier (Muir et al., 2011). It was concluded that CARDS TX can induce allergic inflammation in rodents and in a subset of patients with refractory asthma who are consistently positive for CARDS TX for up to 600 days (Maselli et al., 2018). CARDS TX exacerbated asthma-like inflammation in BALB/c mice through ovalbumin-induced mice models, indicating that CARDS TX can worsen allergic asthma, highlighting the potential importance of CARDS TX in the etiology and exacerbation of human asthma (Medina et al., 2014). CARDS TX can cause ciliostasis, lymphocyte infiltration, increased tissue permeability, and cell death. However, the relationship among CARDS TX, *M. pneumoniae*, and asthma needs to be further investigated.

### ADP-Ribosyltransferase Activity of Community-Acquired Respiratory Distress Syndrome Toxin

ADP-ribosylating toxins play an essential role in the pathogenesis of several bacteria, including *B. pertussis*, *Pseudomonas aeruginosa*, and *C. diphtheriae* (Glowacki et al., 2002; Aravind et al., 2015; Tamamura et al., 2017; Cheng and Wiedmann, 2019). ADP-ribosylation is a post-translational modification that catalyzes the hydrolysis nicotinamide adenine dinucleotide (NAD) and the transfer of an ADP-ribosyl group from NAD^+^ to host cell proteins with the release of nicotinamide (Pallen et al., 2001; Holbourn et al., 2006). In some bacterial toxins, mono ADP-ribosyltransferase catalyzes the ADP-ribosyl group, which is the main cause of host cell cytotoxicity, whereas the poly-ADP-ribosyltransferases catalyze multiple ADP-ribose groups to host cell proteins (Han and Tainer, 2002; Castagnini et al., 2012; Asokanathan et al., 2018). CARDS TX is a mono ADP-ribosyltransferase protein, consisting of three conserved motifs: catalytic glutamate, STS motif, and arginine, which is congruent with the active site motif of the bacterial ADP-ribosylating toxins (Kannan and Baseman, 2006). Several bacterial ADP-ribosylating toxins, such as pertussis toxin, undergo an enzymatic activation after the cleavage of the disulfide bridge (Lai, 1986; Kannan and Baseman, 2006).

Generally, ADP-ribosylating toxins are classified into the *V. cholerae* cholera toxin (CT) and *C. diphtheriae* diphtheria toxin (DT) groups, and the CT group is subsequently divided into *P. aeruginosa* exoenzyme S (ExoS) -like, C2-like, C3-like, and CT-*B. pertussis* pertussis toxin (PT)-like toxins subgroups (Fieldhouse et al., 2010). Although phylogenetic analysis showed that CARDS TX is clustered into the C2-like toxin subgroup, it is generally considered a member of CT-PT-like toxin subgroup, which includes the cholera and pertussis toxins (Fieldhouse et al., 2010).

Importantly, it has been demonstrated that ADPRT activity of CARDS TX transfer of ADP-ribosyl group from NAD^+^ to NLRP3 (Segovia et al., 2018), which is essential for the release of inflammatory factors and subsequent cytopathic effects (Bose et al., 2014; Segovia et al., 2018). Recently, the C3-type ADP-ribosylating toxins from bacteria have become invaluable tools for the studying of G protein-linked receptors (Aktories and Hall, 1989). Interestingly, the majority of *Pseudomonas aeruginosa* strains secrete a bifunctional toxin ExoS with GTPase- and ADP-ribosyltransferase activities, both of which inhibit the internalization of bacteria (Rangel et al., 2014). In summary, CARDS TX is a bifunctional toxin, but whether the ADPRT activity of CARDS TX affects its internalization function has not been clarified.

### Cytoplasmic Vacuolization by Community-Acquired Respiratory Distress Syndrome Toxin

Cytoplasmic vacuolization is a well-known morphological phenomenon observed in mammalian cells induced by bacterial virulence factors (Shubin et al., 2016), which is comprised of various bacterial toxins, such as cytotoxin VacA from *H. pylori* (Cover and Blaser, 1992; Cover and Blanke, 2005), and AB5 subtilase cytotoxin (SubAB) of Shiga-toxigenic *E. coli* (Paton et al., 2004). Studies suggest that rCARDS TX could induce cellular vacuolation, including cytoplasmic and nuclear vacuolization, in most mammalian cell lines, such as CHO, HeLa cells, bronchiolar epithelium cells, and baboon tracheal rings (Kannan and Baseman, 2006; Hardy et al., 2009). The intralumenal environment of rCARDS TX-induced vacuoles was acidic, and the vacuoles were produced from the endocytic pathways (Johnson et al., 2011). Vacuoles induced by CARDS TX are similar to the cytotoxin VacA from *H. pylori* (Cover and Blaser, 1992; Cover and Blanke, 2005).

Cytoplasmic vacuolization of mammalian cells can be transient or irreversible (Shubin et al., 2016). For the CARDS TX-induced cytoplasmic vacuolization, it may be transient but for at least 37 days, after which new cells gradually replace and repair (Hardy et al., 2009). The formation of rCARDS TX-induced vacuoles is inhibited by the vacuolar ATPase inhibitor, bafilomycin A1, and the ionophore, monensin (Hardy et al., 2009). Importantly, rCARDS TX-mediated vacuolization is originated from the perinuclear region, is enriched in markers for late endocytic compartments, and recruits Rab9 from the Rab small GTPase family, LAMP1, and LAMP2, but not Rab7, which is different from VacA, in that its late endosome markers include Rab7, LAMP1, and Lgp110 (Cover and Blanke, 2005; Johnson et al., 2011). Rab7 mediates early to the late endosome, and late endosome to lysosome transport, whereas Rab9 is involved in late endosome to Golgi transport (Pagano, 2003; Kucera et al., 2016). Because there was no increase in the cellular levels of Rab9 in rCARDS TX treated or untreated cells at different time points, indicated that the accumulation of Rab9 around the membranes of rCARDS TX-induced vacuoles was likely due to redistribution and not re-synthesis (Johnson et al., 2011). It is possible that Rab9 can be used as a molecular carrier to regulate intracellular trafficking (Kucera et al., 2016). In addition, Rab9 is also involved in the endosomal transport of lipids (Choudhury et al., 2002). When Rab9 was by dominant-negative, rCARDS TX-induced vacuoles were considerably reduced, but not completely dismissed, indicating that Rab9 plays a key role in *M. pneumoniae* infection (Johnson et al., 2011). However, whether other proteins interact with Rab9 in cellular vacuolization remains unknown.

The N-terminal domain of VacA is responsible for the *H. pylori-induced* “vacuolation”; however, no sequence similarity of the C-terminus of CARDS TX to the functional vacuolating domains of VacA or SubAB have been found yet (Paton et al., 2004; Cover and Blanke, 2005). Normally, vacuolization is often accompanied with cell death; however, the mechanism remains unclear (Shubin et al., 2016). After rCARDS TX treatment, the histopathology normally includes cell vacuolation, marked deterioration of ciliary movement, lymphoplasmacytic infiltrate, and eventually cell death. Vacuolation seems to be particularly relevant in the pathogenic process (Kannan and Baseman, 2006; Kannan et al., 2011). Notably, V-ATPase (vacuolar ATPase) blockers completely inhibited vacuole formation induced by CARDS TX, whereas endosomal pH had less impact on cell vacuolation. V-ATPase plays an important role in CARDS TX-induced vacuole formation (Ramasamy et al., 2021). Further studies are needed to examine the effect of inhibiting vacuolation on CARDS TX toxicity.

## Conclusion and Perspectives

CARDS TX has a dual-function feature, which possesses ADPRT and vacuolation activities (Becker et al., 2015). Structurally, CARDS TX contains special disulfide bonds and retrograde transport KELED motifs, which play important roles in its cytotoxic effects (Ramasamy et al., 2018; Balasubramanian et al., 2019). Findings showed that the pathogenic effect of CARDS TX is influenced by its structural and functional composition (Kannan and Baseman, 2006; Hardy et al., 2009). Recent studies have shown that V-ATPase plays an important role in CARDS TX-induced vacuole formation (Ramasamy et al., 2021). Existing studies have demonstrated that CARDS TX exerts its biological effects through vacuolar ATPase proton pump and host cell endosomal acidic environment, but the mechanism of its endocytosis is unknown (Ramasamy et al., 2021). Further research is needed to determine whether the toxic effects of inhibiting vacuolation on CARDS TX is diminished, and that the repair mechanism of vacuolation is also a good research point. The study on CARDS TX from *M. pneumoniae* could not only have a profound impact on the pathogenesis of *M. pneumoniae*, but also provide a certain research basis for other bacterial toxins in microorganisms, especially bifunctional toxin proteins.

At present, homologous CARDS TX is present in only a few mycoplasmas, some of which still have unexpressed toxin proteins. In the current study, CARDS TX was found to cause an increase in mucus proliferation, and exacerbation of asthma (Medina et al., 2012, 2014), but the pathogenic mechanism between CARDS TX and asthma remains unclear. CARDS TX is positively correlated with the severity of the disease, can even be present in patients for up to 600 days, and possesses a high immunogenic response (Peters et al., 2011; Maselli et al., 2018). However, CARDS TX is still not clinically used as a marker for detecting *M. pneumoniae* infection or as a target for vaccine of *M. pneumoniae*. Although there are still too many problems to be improved thus far, these are an important direction for future research. In summary, the characteristics of CARDS TX need to be further studied and explored to provide certain help for the study of the pathogenic mechanism of *M. pneumoniae*, and development of clinical diagnosis and vaccine. Comprehensive studies should be performed to determine the main pathogenic proteins of *M. pneumoniae* to explore their toxic effects on cells.

## Author Contributions

XS, XY, and JH prepared and wrote the original draft. HL and LC handled the figures design. JH was responsible for the supervision. JH, XY, KL, ZY, and WT provided critical revisions for this review. All authors contributed to the review and approved the submitted manuscript.

## Conflict of Interest

The authors declare that the research was conducted in the absence of any commercial or financial relationships that could be construed as a potential conflict of interest.

## Publisher’s Note

All claims expressed in this article are solely those of the authors and do not necessarily represent those of their affiliated organizations, or those of the publisher, the editors and the reviewers. Any product that may be evaluated in this article, or claim that may be made by its manufacturer, is not guaranteed or endorsed by the publisher.

## References

[B1] Aktories K. Hall A. (1989). Botulinum ADP-ribosyltransferase C3: a new tool to study low molecular weight GTP-binding proteins. *Trends Pharmacol. Sci.* 10 415–418. 10.1016/0165-6147(89)90191-0 2515641

[B2] Ali S. A. Hassan M. I. Islam A. Ahmad F. (2014). A review of methods available to estimate solvent-accessible surface areas of soluble proteins in the folded and unfolded states. *Curr. Protein Pept. Sci.* 15 456–476. 10.2174/1389203715666140327114232 24678666

[B3] Aravind L. Zhang D. de Souza R. F. Anand S. Iyer L. M. (2015). The natural history of ADP-ribosyltransferases and the ADP-ribosylation system. *Curr. Top. Microbiol. Immunol.* 384 3–32. 10.1007/82_2014_414 25027823 PMC6126934

[B4] Asokanathan C. Tierney S. Ball C. R. Buckle G. Day A. Tanley S. (2018). An ELISA method to estimate the mono ADP-ribosyltransferase activities: e.g in pertussis toxin and vaccines. *Anal. Biochem.* 540–541 15–19. 10.1016/j.ab.2017.10.025 29108883

[B5] Atkinson T. P. Balish M. F. Waites K. B. (2008). Epidemiology, clinical manifestations, pathogenesis and laboratory detection of *Mycoplasma pneumoniae* infections. *FEMS Microbiol Rev.* 32 956–973. 10.1111/j.1574-6976.2008.00129.x 18754792

[B6] Atkinson T. P. Waites K. B. (2014). *Mycoplasma pneumoniae* infections in childhood. *Pediatr. Infect. Dis. J.* 33 92–94. 10.1097/inf.0000000000000171 24346598

[B7] Balasubramanian S. Pandranki L. Maupin S. Ramasamy K. Taylor A. B. Hart P. J. (2019). Disulfide bond of *Mycoplasma pneumoniae* community-acquired respiratory distress syndrome toxin is essential to maintain the ADP-ribosylating and vacuolating activities. *Cell. Microbiol.* 21:e13032. 10.1111/cmi.13032 30977272 PMC6612593

[B8] Baseman J. B. Lange M. Criscimagna N. L. Giron J. A. Thomas C. A. (1995). Interplay between mycoplasmas and host target cells. *Microb. Pathog.* 19 105–116. 10.1006/mpat.1995.0050 8577234

[B9] Becker A. Kannan T. R. Taylor A. B. Pakhomova O. N. Zhang Y. Somarajan S. R. (2015). Structure of CARDS toxin, a unique ADP-ribosylating and vacuolating cytotoxin from *Mycoplasma pneumoniae*. *Proc. Natl. Acad. Sci. U.S.A.* 112 5165–5170. 10.1073/pnas.1420308112 25848012 PMC4413325

[B10] Berg C. P. Kannan T. R. Klein R. Gregor M. Baseman J. B. Wesselborg S. (2009). *Mycoplasma* antigens as a possible trigger for the induction of antimitochondrial antibodies in primary biliary cirrhosis. *Liver Int.* 29 797–809. 10.1111/j.1478-3231.2008.01942.x 19638108

[B11] Bose S. Segovia J. A. Somarajan S. R. Chang T. H. Kannan T. R. Baseman J. B. (2014). ADP-ribosylation of NLRP3 by *Mycoplasma pneumoniae* CARDS toxin regulates inflammasome activity. *mBio* 5:e02186-14. 10.1128/mBio.02186-14 25538194 PMC4278538

[B12] Capitani M. Sallese M. (2009). The KDEL receptor: new functions for an old protein. *FEBS Lett.* 583 3863–3871. 10.1016/j.febslet.2009.10.053 19854180

[B13] Castagnini M. Picchianti M. Talluri E. Biagini M. Del Vecchio M. Di Procolo P. (2012). Arginine-specific mono ADP-ribosylation *in vitro* of antimicrobial peptides by ADP-ribosylating toxins. *PLoS One* 7:e41417. 10.1371/journal.pone.0041417 22879887 PMC3413682

[B14] Cheng R. A. Wiedmann M. (2019). The ADP-ribosylating toxins of *Salmonella*. *Toxins (Basel)* 11:416. 10.3390/toxins11070416 31315299 PMC6669713

[B15] Choudhury A. Dominguez M. Puri V. Sharma D. K. Narita K. Wheatley C. L. (2002). Rab proteins mediate Golgi transport of caveola-internalized glycosphingolipids and correct lipid trafficking in Niemann-Pick C cells. *J. Clin. Invest.* 109 1541–1550. 10.1172/JCI15420 12070301 PMC151017

[B16] Chu H. W. Jeyaseelan S. Rino J. G. Voelker D. R. Wexler R. B. Campbell K. (2005). TLR2 signaling is critical for *Mycoplasma pneumoniae*-induced airway mucin expression. *J. Immunol.* 174 5713–5719. 10.4049/jimmunol.174.9.5713 15843573

[B17] Costerton J. W. Stewart P. S. Greenberg E. P. (1999). Bacterial biofilms: a common cause of persistent infections. *Science* 284 1318–1322. 10.1126/science.284.5418.1318 10334980

[B18] Cover T. L. Blanke S. R. (2005). *Helicobacter pylori* VacA, a paradigm for toxin multifunctionality. *Nat. Rev. Microbiol.* 3 320–332. 10.1038/nrmicro1095 15759043

[B19] Cover T. L. Blaser M. J. (1992). Purification and characterization of the vacuolating toxin from *Helicobacter pylori*. *J. Biol. Chem.* 267 10570–10575. 1587837

[B20] Dallacasagrande V. Hajjar K. A. (2020). Annexin A2 in inflammation and host defense. *Cells* 9:1499. 10.3390/cells9061499 32575495 PMC7348701

[B21] Dy A. Tanyaratsrisakul S. Voelker D. R. Ledford J. G. (2018). The emerging roles of surfactant protein-A in asthma. *J. Clin. Cell. Immunol.* 9:553. 10.4172/2155-9899.1000553 30123671 PMC6092951

[B22] Feng M. Schaff A. C. Balish M. F. (2020). *Mycoplasma pneumoniae* biofilms grown *in vitro*: traits associated with persistence and cytotoxicity. *Microbiology (Reading)* 166 629–640. 10.1099/mic.0.000928 32421492

[B23] Fieldhouse R. J. Turgeon Z. White D. Merrill A. R. (2010). Cholera- and anthrax-like toxins are among several new ADP-ribosyltransferases. *PLoS Comput. Biol.* 6:e1001029. 10.1371/journal.pcbi.1001029 21170356 PMC3000352

[B24] Glowacki G. Braren R. Firner K. Nissen M. Kühl M. Reche P. (2002). The family of toxin-related ecto-ADP-ribosyltransferases in humans and the mouse. *Protein Sci.* 11 1657–1670. 10.1110/ps.0200602 12070318 PMC2373659

[B25] Grindheim A. K. Saraste J. Vedeler A. (2017). Protein phosphorylation and its role in the regulation of Annexin A2 function. *Biochim. Biophys. Acta Gen. Subj.* 1861(11 Pt A) 2515–2529. 10.1016/j.bbagen.2017.08.024 28867585

[B26] Groslambert M. Py B. F. (2018). Spotlight on the NLRP3 inflammasome pathway. *J. Inflamm. Res.* 11 359–374. 10.2147/jir.s141220 30288079 PMC6161739

[B27] Großhennig S. Ischebeck T. Gibhardt J. Busse J. Feussner I. Stülke J. (2016). Hydrogen sulfide is a novel potential virulence factor of *Mycoplasma pneumoniae*: characterization of the unusual cysteine desulfurase/desulfhydrase HapE. *Mol. Microbiol.* 100 42–54. 10.1111/mmi.13300 26711628

[B28] Han S. Tainer J. A. (2002). The ARTT motif and a unified structural understanding of substrate recognition in ADP-ribosylating bacterial toxins and eukaryotic ADP-ribosyltransferases. *Int. J. Med. Microbiol.* 291 523–529. 10.1078/1438-4221-00162 11890553

[B29] Hardy R. D. Coalson J. J. Peters J. Chaparro A. Techasaensiri C. Cantwell A. M. (2009). Analysis of pulmonary inflammation and function in the mouse and baboon after exposure to *Mycoplasma pneumoniae* CARDS toxin. *PLoS One* 4:e7562. 10.1371/journal.pone.0007562 19859545 PMC2762541

[B30] He J. Liu M. Ye Z. Tan T. Liu X. You X. (2016). Insights into the pathogenesis of *Mycoplasma pneumoniae* (Review). *Mol. Med. Rep.* 14 4030–4036. 10.3892/mmr.2016.5765 27667580 PMC5101875

[B31] Holbourn K. P. Shone C. C. Acharya K. R. (2006). A family of killer toxins. Exploring the mechanism of ADP-ribosylating toxins. *FEBS J.* 273 4579–4593. 10.1111/j.1742-4658.2006.05442.x 16956368

[B32] Hooftman A. Angiari S. Hester S. Corcoran S. E. Runtsch M. C. Ling C. (2020). The immunomodulatory metabolite itaconate modifies NLRP3 and inhibits inflammasome activation. *Cell Metab.* 32 468–478.e7. 10.1016/j.cmet.2020.07.016 32791101 PMC7422798

[B33] Jackson M. E. Simpson J. C. Girod A. Pepperkok R. Roberts L. M. Lord J. M. (1999). The KDEL retrieval system is exploited by *Pseudomonas* exotoxin A, but not by Shiga-like toxin-1, during retrograde transport from the Golgi complex to the endoplasmic reticulum. *J. Cell Sci.* 112 (Pt 4) 467–475. 10.1242/jcs.112.4.467 9914159

[B34] Jia J. Yue X. Zhu L. Jing S. Wang Y. Gim B. (2021). KDEL receptor is a cell surface receptor that cycles between the plasma membrane and the Golgi *via* clathrin-mediated transport carriers. *Cell. Mol. Life Sci.* 78 1085–1100. 10.1007/s00018-020-03570-3 32562023 PMC11072833

[B35] Johnson C. Kannan T. R. Baseman J. B. (2009). Characterization of a unique ADP-ribosyltransferase of *Mycoplasma penetrans*. *Infect. Immun.* 77 4362–4370. 10.1128/IAI.00044-09 19651868 PMC2747967

[B36] Johnson C. Kannan T. R. Baseman J. B. (2011). Cellular vacuoles induced by *Mycoplasma pneumoniae* CARDS toxin originate from Rab9-associated compartments. *PLoS One* 6:e22877. 10.1371/journal.pone.0022877 21829543 PMC3146493

[B37] Kannan T. R. Baseman J. B. (2006). ADP-ribosylating and vacuolating cytotoxin of *Mycoplasma pneumoniae* represents unique virulence determinant among bacterial pathogens. *Proc. Natl. Acad. Sci. U.S.A.* 103 6724–6729. 10.1073/pnas.0510644103 16617115 PMC1458948

[B38] Kannan T. R. Coalson J. J. Cagle M. Musatovova O. Hardy R. D. Baseman J. B. (2011). Synthesis and distribution of CARDS toxin during Mycoplasma pneumoniae infection in a murine model. *J. Infect. Dis.* 204 1596–1604. 10.1093/infdis/jir557 21957154 PMC3222103

[B39] Kannan T. R. Hardy R. D. Coalson J. J. Cavuoti D. C. Siegel J. D. Cagle M. (2012). Fatal outcomes in family transmission of *Mycoplasma pneumoniae*. *Clin. Infect. Dis.* 54 225–231. 10.1093/cid/cir769 22052890 PMC3245726

[B40] Kannan T. R. Krishnan M. Ramasamy K. Becker A. Pakhomova O. N. Hart P. J. (2014). Functional mapping of community-acquired respiratory distress syndrome (CARDS) toxin of *Mycoplasma pneumoniae* defines regions with ADP-ribosyltransferase, vacuolating and receptor-binding activities. *Mol. Microbiol.* 93 568–581. 10.1111/mmi.12680 24948331 PMC4116743

[B41] Kannan T. R. Musatovova O. Balasubramanian S. Cagle M. Jordan J. L. Krunkosky T. M. (2010). *Mycoplasma pneumoniae* community acquired respiratory distress syndrome toxin expression reveals growth phase and infection-dependent regulation. *Mol. Microbiol.* 76 1127–1141. 10.1111/j.1365-2958.2010.07092.x 20199607 PMC2883071

[B42] Kannan T. R. Provenzano D. Wright J. R. Baseman J. B. (2005). Identification and characterization of human surfactant protein A binding protein of *Mycoplasma pneumoniae*. *Infect. Immun.* 73 2828–2834. 10.1128/IAI.73.5.2828-2834.2005 15845487 PMC1087375

[B43] Khan I. Steeg P. S. (2021). Endocytosis: a pivotal pathway for regulating metastasis. *Br. J. Cancer* 124 66–75. 10.1038/s41416-020-01179-8 33262521 PMC7782782

[B44] Kraft M. Adler K. B. Ingram J. L. Crews A. L. Atkinson T. P. Cairns C. B. (2008). *Mycoplasma pneumoniae* induces airway epithelial cell expression of MUC5AC in asthma. *Eur. Respir. J.* 31 43–46. 10.1183/09031936.00103307 18166592

[B45] Krishnan M. Kannan T. R. Baseman J. B. (2013). *Mycoplasma pneumoniae* CARDS toxin is internalized *via* clathrin-mediated endocytosis. *PLoS One* 8:e62706. 10.1371/journal.pone.0062706 23667510 PMC3647021

[B46] Kucera A. Borg Distefano M. Berg-Larsen A. Skjeldal F. Repnik U. Bakke O. (2016). Spatiotemporal resolution of Rab9 and CI-MPR dynamics in the endocytic pathway. *Traffic* 17 211–229. 10.1111/tra.12357 26663757

[B47] Kumar S. Roy R. D. Sethi G. R. Saigal S. R. (2019). *Mycoplasma pneumoniae* infection and asthma in children. *Trop. Doct.* 49 117–119. 10.1177/0049475518816591 30537911

[B48] Lai C. Y. (1986). Bacterial protein toxins with latent ADP-ribosyl transferases activities. *Adv. Enzymol. Relat. Areas Mol. Biol.* 58 99–140. 10.1002/9780470123041.ch3 3012972

[B49] Landeta C. McPartland L. Tran N. Q. Meehan B. M. Zhang Y. Tanweer Z. (2019). Inhibition of *Pseudomonas aeruginosa* and *Mycobacterium tuberculosis* disulfide bond forming enzymes. *Mol. Microbiol.* 111 918–937. 10.1111/mmi.14185 30556355 PMC6458069

[B50] Lee K. Y. (2008). Pediatric respiratory infections by *Mycoplasma pneumoniae*. *Expert Rev. Anti Infect. Ther.* 6 509–521. 10.1586/14787210.6.4.509 18662117

[B51] Li G. Fan L. Wang Y. Huang L. Wang M. Zhu C. (2019). High co-expression of TNF-α and CARDS toxin is a good predictor for refractory *Mycoplasma pneumoniae* pneumonia. *Mol. Med.* 25:38. 10.1186/s10020-019-0105-2 31399022 PMC6688229

[B52] Lluch-Senar M. Cozzuto L. Cano J. Delgado J. Llórens-Rico V. Pereyre S. (2015a). Comparative “-omics” in *Mycoplasma pneumoniae* clinical isolates reveals key virulence factors. *PLoS One* 10:e0137354. 10.1371/journal.pone.0137354 26335586 PMC4559472

[B53] Lluch-Senar M. Delgado J. Chen W. H. Lloréns-Rico V. O’Reilly F. J. Wodke J. A. (2015b). Defining a minimal cell: essentiality of small ORFs and ncRNAs in a genome-reduced bacterium. *Mol. Syst. Biol.* 11:780. 10.15252/msb.20145558 25609650 PMC4332154

[B54] Luo H. He J. Qin L. Chen Y. Chen L. Li R. (2021). *Mycoplasma pneumoniae* lipids license TLR-4 for activation of NLRP3 inflammasome and autophagy to evoke a proinflammatory response. *Clin. Exp. Immunol.* 203 66–79. 10.1111/cei.13510 32894580 PMC7744503

[B55] Maselli D. J. Medina J. L. Brooks E. G. Coalson J. J. Kannan T. R. Winter V. T. (2018). The immunopathologic effects of *Mycoplasma pneumoniae* and community-acquired respiratory distress syndrome toxin. A primate model. *Am. J. Respir. Cell Mol. Biol.* 58 253–260. 10.1165/rcmb.2017-0006OC 28915064 PMC5805996

[B56] McMahon H. T. Boucrot E. (2011). Molecular mechanism and physiological functions of clathrin-mediated endocytosis. *Nat. Rev. Mol. Cell Biol.* 12 517–533. 10.1038/nrm3151 21779028

[B57] Medina J. L. Brooks E. G. Chaparro A. Dube P. H. (2017). *Mycoplasma pneumoniae* CARDS toxin elicits a functional IgE response in Balb/c mice. *PLoS One* 12:e0172447. 10.1371/journal.pone.0172447 28199385 PMC5310781

[B58] Medina J. L. Coalson J. J. Brooks E. G. Le Saux C. J. Winter V. T. Chaparro A. (2014). *Mycoplasma pneumoniae* CARDS toxin exacerbates ovalbumin-induced asthma-like inflammation in BALB/c mice. *PLoS One* 9:e102613. 10.1371/journal.pone.0102613 25058417 PMC4109942

[B59] Medina J. L. Coalson J. J. Brooks E. G. Winter V. T. Chaparro A. Principe M. F. (2012). *Mycoplasma pneumoniae* CARDS toxin induces pulmonary eosinophilic and lymphocytic inflammation. *Am. J. Respir. Cell Mol. Biol.* 46 815–822. 10.1165/rcmb.2011-0135OC 22281984 PMC3380286

[B60] Muir M. T. Cohn S. M. Louden C. Kannan T. R. Baseman J. B. (2011). Novel toxin assays implicate *Mycoplasma pneumoniae* in prolonged ventilator course and hypoxemia. *Chest* 139 305–310. 10.1378/chest.10-1222 20884727 PMC3032367

[B61] Pagano R. E. (2003). Endocytic trafficking of glycosphingolipids in sphingolipid storage diseases. *Philos. Trans. R. Soc. Lond. B Biol. Sci.* 358 885–891. 10.1098/rstb.2003.1275 12803922 PMC1693187

[B62] Pakhomova O. N. Taylor A. B. Becker A. Holloway S. P. Kannan T. R. Baseman J. B. (2010). Crystallization of community-acquired respiratory distress syndrome toxin from *Mycoplasma pneumoniae*. *Acta Crystallogr. Sect. F Struct. Biol. Cryst. Commun.* 66 (Pt 3) 294–296. 10.1107/S1744309110000114 20208164 PMC2833040

[B63] Pallen M. J. Lam A. C. Loman N. J. McBride A. (2001). An abundance of bacterial ADP-ribosyltransferases–implications for the origin of exotoxins and their human homologues. *Trends Microbiol.* 9 302–307; discussion 308. 10.1016/s0966-842x(01)02074-1 11435081

[B64] Parrott G. L. Kinjo T. Fujita J. A. (2016). Compendium for *Mycoplasma pneumoniae*. *Front. Microbiol.* 7:513. 10.3389/fmicb.2016.00513 27148202 PMC4828434

[B65] Paton A. W. Srimanote P. Talbot U. M. Wang H. Paton J. C. (2004). A new family of potent AB(5) cytotoxins produced by Shiga toxigenic *Escherichia coli*. *J. Exp. Med.* 200 35–46. 10.1084/jem.20040392 15226357 PMC2213318

[B66] Pereira C. Rodrigues I. S. Pereira L. Lisboa J. Pinto R. D. Araújo L. (2020). Role of AIP56 disulphide bond and its reduction by cytosolic redox systems for efficient intoxication. *Cell Microbiol.* 22:e13109. 10.1111/cmi.13109 31454143

[B67] Peters J. Singh H. Brooks E. G. Diaz J. Kannan T. R. Coalson J. J. (2011). Persistence of community-acquired respiratory distress syndrome toxin-producing *Mycoplasma pneumoniae* in refractory asthma. *Chest* 140 401–407. 10.1378/chest.11-0221 21622549 PMC3148797

[B68] Piboonpocanun S. Chiba H. Mitsuzawa H. Martin W. Murphy R. C. Harbeck R. J. (2005). Surfactant protein A binds *Mycoplasma pneumoniae* with high affinity and attenuates its growth by recognition of disaturated phosphatidylglycerols. *J. Biol. Chem.* 280 9–17. 10.1074/jbc.M411570200 15498759

[B69] Pritchard R. E. Balish M. F. (2015). *Mycoplasma iowae*: relationships among oxygen, virulence, and protection from oxidative stress. *Vet. Res.* 46:36. 10.1186/s13567-015-0170-7 25880161 PMC4367981

[B70] Ramasamy K. Balasubramanian S. Kirkpatrick A. Szabo D. Pandranki L. Baseman J. B. (2021). *Mycoplasma pneumoniae* CARDS toxin exploits host cell endosomal acidic pH and vacuolar ATPase proton pump to execute its biological activities. *Sci. Rep.* 11:11571. 10.1038/s41598-021-90948-3 34078958 PMC8172646

[B71] Ramasamy K. Balasubramanian S. Manickam K. Pandranki L. Taylor A. B. Hart P. J. (2018). *Mycoplasma pneumoniae* community-acquired respiratory distress syndrome toxin uses a novel KELED sequence for retrograde transport and subsequent cytotoxicity. *mBio* 9:e01663-17. 10.1128/mBio.01663-17 29362229 PMC5784248

[B72] Rangel S. M. Logan L. K. Hauser A. R. (2014). The ADP-ribosyltransferase domain of the effector protein ExoS inhibits phagocytosis of *Pseudomonas aeruginosa* during pneumonia. *mBio* 5:e01080-14. 10.1128/mBio.01080-14 24917597 PMC4056551

[B73] Rottem S. (2003). Interaction of mycoplasmas with host cells. *Physiol. Rev.* 83 417–432. 10.1152/physrev.00030.2002 12663864

[B74] Royal J. M. Reeves M. A. Matoba N. (2019). Repeated oral administration of a KDEL-tagged recombinant cholera toxin B subunit effectively mitigates DSS colitis despite a robust immunogenic response. *Toxins (Basel)* 11:678. 10.3390/toxins11120678 31756977 PMC6950078

[B75] Sandvig K. Grimmer S. Iversen T. G. Rodal K. Torgersen M. L. Nicoziani P. (2000). Ricin transport into cells: studies of endocytosis and intracellular transport. *Int. J. Med. Microbiol.* 290 415–420. 10.1016/S1438-4221(00)80055-7 11111920

[B76] Segovia J. A. Chang T. H. Winter V. T. Coalson J. J. Cagle M. P. Pandranki L. (2018). NLRP3 is a critical regulator of inflammation and innate immune cell response during *Mycoplasma pneumoniae* infection. *Infect. Immun.* 86:e00548-17. 10.1128/IAI.00548-17 29061706 PMC5736809

[B77] Shimizu T. (2016). Inflammation-inducing factors of *Mycoplasma pneumoniae*. *Front. Microbiol.* 7:414. 10.3389/fmicb.2016.00414 27065977 PMC4814563

[B78] Shimizu T. Kimura Y. Kida Y. Kuwano K. Tachibana M. Hashino M. (2014). Cytadherence of *Mycoplasma pneumoniae* induces inflammatory responses through autophagy and toll-like receptor 4. *Infect. Immun.* 82 3076–3086. 10.1128/IAI.01961-14 24799628 PMC4097642

[B79] Shubin A. V. Demidyuk I. V. Komissarov A. A. Rafieva L. M. Kostrov S. V. (2016). Cytoplasmic vacuolization in cell death and survival. *Oncotarget* 7 55863–55889. 10.18632/oncotarget.10150 27331412 PMC5342458

[B80] Simmons W. L. Daubenspeck J. M. Osborne J. D. Balish M. F. Waites K. B. Dybvig K. (2013). Type 1 and type 2 strains of *Mycoplasma pneumoniae* form different biofilms. *Microbiology (Reading)* 159 (Pt 4) 737–747. 10.1099/mic.0.064782-0 23412845 PMC4036059

[B81] Somarajan S. R. Al-Asadi F. Ramasamy K. Pandranki L. Baseman J. B. Kannan T. R. (2014). Annexin A2 mediates *Mycoplasma pneumoniae* community-acquired respiratory distress syndrome toxin binding to eukaryotic cells. *mBio* 5:e01497-14. 10.1128/mBio.01497-14 25139904 PMC4147866

[B82] Tamamura Y. Tanaka K. Uchida I. (2017). Characterization of pertussis-like toxin from *Salmonella* spp. that catalyzes ADP-ribosylation of G proteins. *Sci. Rep.* 7:2653. 10.1038/s41598-017-02517-2 28572615 PMC5454059

[B83] Techasaensiri C. Tagliabue C. Cagle M. Iranpour P. Katz K. Kannan T. R. (2010). Variation in colonization, ADP-ribosylating and vacuolating cytotoxin, and pulmonary disease severity among mycoplasma pneumoniae strains. *Am. J. Respir. Crit. Care Med.* 182 797–804. 10.1164/rccm.201001-0080OC 20508214 PMC2949405

[B84] Waites K. B. Talkington D. F. (2004). *Mycoplasma pneumoniae* and its role as a human pathogen. *Clin. Microbiol. Rev.* 17 697–728. 10.1128/CMR.17.4.697-728.2004 15489344 PMC523564

[B85] Wang Z. Bao H. Liu Y. Wang Y. Qin J. Yang L. (2020). Interleukin-23 derived from CD16+ monocytes drives IL-17 secretion by TLR4 pathway in children with *Mycoplasma pneumoniae* pneumonia. *Life Sci.* 258:118149. 10.1016/j.lfs.2020.118149 32726660

[B86] Watanabe H. Uruma T. Nakamura H. Aoshiba K. (2014). The role of *Mycoplasma pneumoniae* infection in the initial onset and exacerbations of asthma. *Allergy Asthma Proc.* 35 204–210. 10.2500/aap.2014.35.3742 24801462

[B87] Xiao L. Ptacek T. Osborne J. D. Crabb D. M. Simmons W. L. Lefkowitz E. J. (2015). Comparative genome analysis of *Mycoplasma pneumoniae*. *BMC Genomics* 16:610. 10.1186/s12864-015-1801-0 26275904 PMC4537597

[B88] Xue G. Zhao H. Yan C. Li S. Cui J. Feng Y. (2021). Evaluation of the CARDS toxin and its fragment for the serodiagnosis of *Mycoplasma pneumoniae* infections. *Eur. J. Clin. Microbiol. Infect. Dis.* 40 1705–1711. 10.1007/s10096-021-04209-2 33733396

[B89] Youn Y. S. Lee K. Y. (2012). *Mycoplasma pneumoniae* pneumonia in children. *Korean J. Pediatr.* 55 42–47. 10.3345/kjp.2012.55.2.42 22375148 PMC3286761

